# Killing cancer with platycodin D through multiple mechanisms

**DOI:** 10.1111/jcmm.12749

**Published:** 2015-12-09

**Authors:** Muhammad Khan, Amara Maryam, He Zhang, Tahir Mehmood, Tonghui Ma

**Affiliations:** ^1^ College of Basic Medical SciencesDalian Medical UniversityDalianLiaoningChina

**Keywords:** platycodin D, saponin, apoptosis, autophagy, invasion, metastasis

## Abstract

Cancer is a multi‐faceted disease comprised of a combination of genetic, epigenetic, metabolic and signalling aberrations which severely disrupt the normal homoeostasis of cell growth and death. Rational developments of highly selective drugs which specifically block only one of the signalling pathways have been associated with limited therapeutic success. Multi‐targeted prevention of cancer has emerged as a new paradigm for effective anti‐cancer treatment. Platycodin D, a triterpenoid saponin, is one the major active components of the roots of *Platycodon grandiflorum* and possesses multiple biological and pharmacological properties including, anti‐nociceptive, anti‐atherosclerosis, antiviral, anti‐inflammatory, anti‐obesity, immunoregulatory, hepatoprotective and anti‐tumour activities. Recently, the anti‐cancer activity of platycodin D has been extensively studied. The purpose of this review was to give our perspectives on the current status of platycodin D and discuss its anti‐cancer activity and molecular mechanisms which may help the further design and conduct of pre‐clinical and clinical trials to develop it successfully into a potential lead drug for oncological therapy. Platycodin D has been shown to fight cancer by inducing apoptosis, cell cycle arrest, and autophagy and inhibiting angiogenesis, invasion and metastasis by targeting multiple signalling pathways which are frequently deregulated in cancers suggesting that this multi‐target activity rather than a single effect may play an important role in developing platycodin D into potential anti‐cancer drug.

## Introduction

Cancer remains one of the major public health problems in both developed and developing countries and represents second leading cause of death worldwide 1, with approximately 14 million new cases and 8.2 million cancer‐related deaths in 2012 1, 2. According to World Health Organization estimate, by 2050, 17.5 million cancer deaths are projected to occur in the world 3 Regardless of the factors contributing to the initiation and development of a particular cancer, accumulating research evidence suggests that most cancers are caused by dysfunction of many genes coding for proteins such as anti‐apoptotic proteins, inhibitors of apoptosis, transcriptional factors, growth factors, growth factor receptors and tumour suppressors, which constituted the targets for cancer treatment 1, 4.

Prevailing chemotherapeutic drugs have limited therapeutic success in cancer; because they are highly toxic, expensive and activate the alternative cell signalling pathways 5. Rational development of highly selective drugs that specifically block only one of the signalling pathways such as monoclonal antibodies that kill the cancer cells by specifically binding to the extracellular domains of receptor tyrosine kinase have been associated with sporadic responses because of emergence of secondary drug resistance 1, 6, 7, 8. As cancer development is a multi‐step process which is characterized by multiple abnormalities rather than a single mutation, it is unlikely to inhibit cancer effectively using mono‐target agent 9, 10. A plethora of research reports have shown that unlike pharmaceutical drugs that act as mono‐target molecules, plants have multi‐target molecules that can regulate cancer growth and progression by interfering with multiple mechanisms which are central to cancer progression 5.

Cancer chemoprevention by natural compound has emerged as a promising and pragmatic approach to reduce the risk of cancer and is gaining increasing attention because it is considered as safe, cost effective and alternative form of modern health care system 4, 11, 12. Plants have a long history of use in the treatment of cancer and remain the most attractive source of anti‐cancer drugs as tremendous chemical diversity is found in millions of plant species 4, 13, 14, 15. There exist several solid reasons for continued interest in exploring plants extensively as a source of new potential therapeutic anti‐cancer agents. First, at present more than 60% FDA approved commercially available anti‐cancer drugs are derived from natural sources including plants 16. However, the plants which have been thoroughly investigated for their potential values as a source of drugs make a very small fraction (about 10%) of the total estimated 2.5 to 5 million plant species 17, 18. As a very small fraction of available plant flora has obviously contributed for more than 60% of prevailing chemotherapeutic anti‐cancer drugs, the need for the identification of novel structures from remaining flora and understanding their molecular mechanisms for the development of more effective therapeutic drugs to reduce the burden of cancer is therefore paramount. Second, nature has spent over 3 billion years to create a wonderful and near‐perfect complex compound library by performing countless high throughput screens to remove inactive and retain bioactive molecules that no synthetic chemist could ever dream up 19. Although combinatorial chemistry is one of the most important methodologies developed by chemists to create new leads in anti‐cancer drug discovery, however, only one FDA approved anti‐cancer drug ‘Sorafenib’ has been made available in public domain by this method from 1981 to 2006 20. Third, a comparative genomic analysis revealed that 70% of cancer‐related human genes have orthologues in *Arabidopsis thaliana* indicating that human and plants use similar receptors and signalling pathways in some cases 21, 22. Given the similarity of many human and plant genes, it can be assumed that some secondary metabolites produced by plants to modulate their own metabolism might be able to interact with molecules which have a role in human cancers. One such example of such a case is multidrug resistance‐like proteins that are used by *Arabidopsis thaliana* to transport auxin have orthlogues in human that play important role in development of multidrug resistance by transporting anti‐cancer drugs out of cells. Flavonoids, which are auxin distribution modulators in Arabidopsis, have been shown to overcome multidrug resistance by modulating p‐glycoprotein in various human cancers 23. Fourth, natural anti‐cancer compounds fit into mechanism‐based approach as perfectly as a hand fits into a glove. There is solid evidence that plant‐derived bioactive compounds inhibit cancer by interfering with multiple mechanisms which are central to cancer progression 1. Fifth, plants often produce bioactive compounds that exceed the current capacity of synthetic chemistry 24, 25.

Plants' secondary metabolites such as alkaloids, terpenes and polyphenols have now been well characterized for their anti‐cancer activity 16, 26, 27, 28, 29, 30. Till now, more than 55,000 terpenes have been isolated 31 but only a small fraction of terpenes is well characterized for their potential value as a source of anti‐cancer drugs 32. Therefore, it is imperative to identify and characterize more and more bioactive compounds with promising anti‐cancer activity for faster development of more effective anti‐cancer drugs. Diterpenoids, sesquiterpene lactones and saponins are the major classes of terpenes with well‐documented anti‐cancer activity against a wide range of human cancers 32, 33, 34. Saponins are a large and structurally diverse group of bioactive compounds 32 which are reported to occur in more than 100 families of plants 35. Chemically, saponins are natural glycosides of steroid or triterpene 36. The steroidal saponins are important precursor molecules for various steroid drugs such as anti‐inflammatory drugs, androgen, oestrogen and progestins 37. The triterpene saponins have been shown to exhibit a wide range of biological and pharmacological activities including anti‐inflammatory, antiviral, anti‐fungal, antibacterial, antioxidant, anti‐cancer, anti‐diarrheal, anti‐ulcerogenic, anti‐oxytocic, antitussive, anticoagulant, immunomodulatory, hepatoprotective, neuroprotective, expectorant, analgesic, molluscicidal, hypoglycaemic and hypocholesterolaemic 36, 37. Modern research has shown that saponins are potential anti‐cancer agents with different mechanisms of action 35. To date, about 150 kinds of natural saponins have been found to possess significant anti‐cancer activity against various human cancer cells 35. Saponins are usually considered toxic because of their haemolytic properties. The number of sugar chains and type of sugar residue attached to C‐3 and C‐28 of aglycone play important role in pore formation as well as in adjuvant activity of triterpenoid saponins. Saponins with two sugar chains induce less haemolytic activity and membrane permeability than those with only one side chain 38, 39. Other studies show that presence of single glucosyl group in sugar chain attached to C‐3 of aglycone is responsible for strong adjuvant property of saponins 40, 41. Platycodin D, an oleanane type triterpenoid saponin possesses two sugar chains; a single glucosyl group attached at position C‐3 and other chain of four sugar residues at position C‐28 of aglycone. Platycodin D has been shown to exert anti‐tumour activity in various cancer cells without affecting normal cells in various *in vitro* 42 and *in vivo* 43, 44, 45 models. Platycodin D has been extensively investigated and found to exhibit significant anti‐cancer activity against a wide range of human cancers types both *in vitro* and *in vivo* through multiple mechanisms which are considered to be crucial and central to cancer development and metastasis. Among other oleanane type, triterpenoid saponins with two sugar chains at position C‐3 and C‐28, tubeimoside I, a bioactive component of Traditional Chinese Medicinal herb *Bolbostemma paniculatum,* has been extensively studied for its anti‐cancer activity against various human cancers including lung cancer, gastric cancer, ovarian cancer, choriocarcinoma cancer, glioma, hepatoma, squamous oesophageal carcinoma and cervical carcinoma 46. The anti‐cancer activity of tubeimoside I has been reported to be associated with apoptosis induction through mitochondrial dysfunction, Bcl‐2 family protein modulation, COX‐2 down‐regulation, caspases activation, reactive oxygen species (ROS) generation, endoplasmic reticulum stress 46, 47, Fas and FasL activation, c‐Jun N‐terminal kinase (JNK) phosphorylation, down‐regulation of tumour necrosis factor‐α, and NF‐κB 48, p38 phosphorylation, Protein kinase B (AKT) and Extracellular Signal Regulating Kinase (ERK) dephosphorylation 49 and G2/M phase arrest by inhibiting tubuline polymerization 50 and down‐regulating the expression of cyclin B1/cdc2 complex through p21 activation 51. The IC_50_ values of tubeimoside I against various cancer cells were comparable to platycodin D. The aim of this review was to summarize the current knowledge and discuss the natural sources, anti‐cancer activity, molecular targets, mechanisms of action and usefulness of platycodin D for anti‐cancer drug development.

## Natural sources

Platycodin D is one of the major bioactive components of the Traditional Chinese Medicinal herb *Platycodon grandiflorum* 52, 53 which is called Jiegeng in Chinese 45 and Chinese balloon flower or common balloon flower in English 37. It is abundantly found in China, Japan and Korea 43, 54. The content of platycodin D in root and aerial parts of *P. grandiflorum* is determined to be 0.20% 52 and 0.018% 53 of dry weight respectively. It can also be obtained from platycoside E and platycodin D3 by enzymatic transformation 55. The yield of platycodin D by enzymatic transformation method is twofold as compared to direct isolation and purification from methanol extract of roots 55. The root of *P. grandiflorum* called Platycodonis Radix 56 or Platycodi Radix 44, 57 is a well‐known Traditional Chinese Medicine used as an expectorant for pulmonary diseases and a remedy for respiratory disorders including cough, colds, sore throat, tonsillitis and chest congestion 54, 58. In Korea, in addition to treating diabetes and respiratory and inflammatory diseases, the roots of *P. grandiflorum* are used to prepare salad and many other traditional Korean dishes. Pan fried roots are also served as a popular dish in Korean traditional foods 37.

## Killing cancer with platycodin D

**Figure 1:**
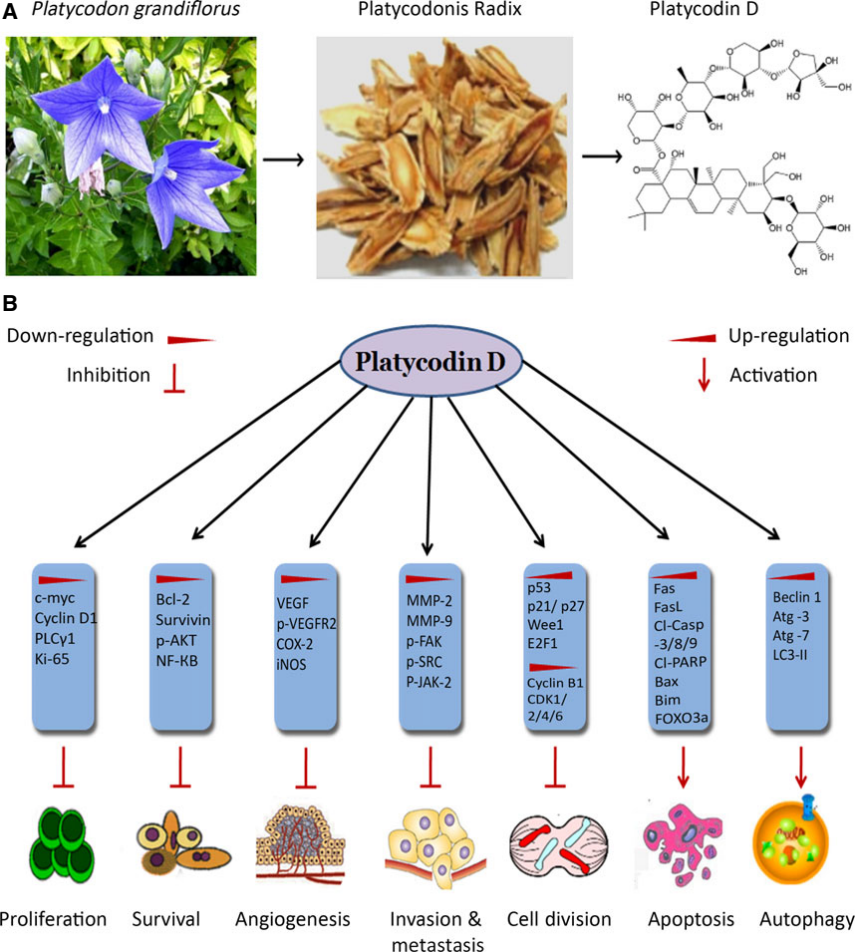
(**A**) Chemical structure and natural source of platycodin D. (**B**), Platycodin D inhibits cancer progression and development by inhibiting proliferation, survival, angiogenesis, invasion, metastasis and cell cycle progression, and ultimately inducing apoptosis and autophagy by modulating expressions of various genes.

Platycodin D (Fig. 1A) is a potential anti‐cancer compound that has been shown to exhibit a broad spectrum of cytotoxicity against a wide range of human cancer cell lines of different origin both *in vitro* and *in vivo* through multiple mechanisms of action involving proliferation and survival inhibition, apoptosis induction, non‐specified apoptotic cell death stimulation, cell cycle arrest, autophagy, and angiogenesis and metastasis inhibition and transcription factors regulation as shown in Figure 1B.

## Targeting apoptosis pathways in cancer cells with platycodin D

Apoptosis is a highly synchronized gene‐controlled process during which a series of intracellular events come into play to decommission the unwanted and cancerous cells 1, 59. It is characterized by typical morphological and biochemical hallmarks, including membrane blebbing, DNA fragmentation, cell shrinkage and caspase‐3 activation 60, 61, 62. Apoptosis is of widespread biological significance playing a vital role in a myriad of physiological and pathological processes in a wide variety of tissues. Apoptosis plays a key role in maintaining tissue homoeostasis by selectively eliminating unwanted or damaged cells. Tissue homoeostasis is regulated through a precise balance between apoptosis and cell proliferation. However, disruption of this balance between apoptosis and cell proliferation may promote severe pathological conditions including, tumourigenesis, neurodegeneration, auto‐immune diseases and developmental abnormalities 63, 64, 65, 66. Inhibition of apoptosis may lead to tumour development and drug resistance 65, 67. Tumour cells use a variety of molecular mechanisms to suppress apoptosis 68. Thus, activation of the molecular mechanisms that regulate the apoptotic machinery in various human malignancies may provide a novel opportunity for cancer drug development. Identification of novel therapeutic agent that have potential to selectively induce apoptosis in cancer cells are the focus of modern anti‐cancer drug discovery.

**Figure 2:**
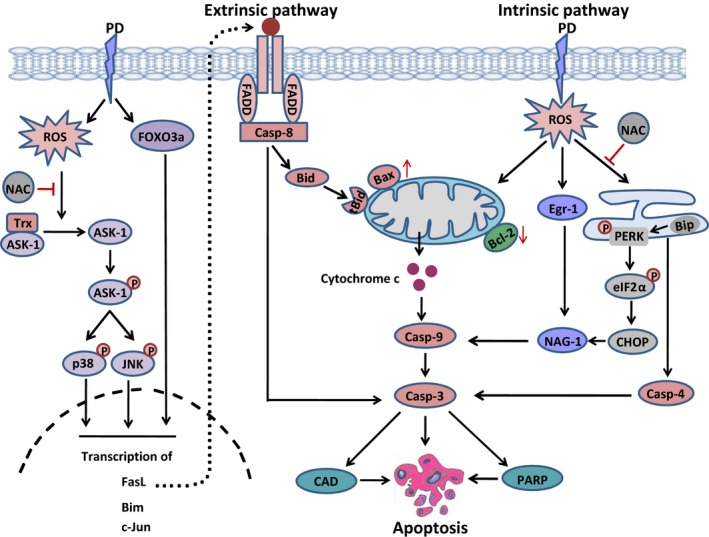
A schematic model of platycodin D‐induced apoptosis in cancer cells. Platycodin D induces ROS generation and increases the expression of transcriptional factor FOXO3a. ROS activates ASK‐1 by reducing the expression of thioredoxin. Once activated, ASK‐1 activates p38 and JNK by phosphorylation. Transcriptional factor FOXO3a and activated p38 and JNK triggered both extrinsic and extrinsic apoptosis by inducing the transcription of apoptosis related genes such as FasL, Bim, c‐Jun. Moreover, platycodin D induces intrinsic apoptosis by modulating the expression of Bcl‐2 family proteins and mitochondrial dysfunction, inducing endoplasmic reticulum stress, increasing the expressions of Egr‐1 and NAG‐1 which results in caspase‐3 activation. Caspase‐3 then executes apoptotic cell death by cleaving PARP and activating CAD.

Platycodin D, a triterpenoid saponin has recently been evaluated for its potential value as a source of anti‐cancer drug. Accumulating evidence from various studies has shown that platycodin D holds the promise to trigger apoptosis to set cancer cells on the road to ruin. Multiple mechanisms have been implicated in apoptosis inducing action of platycodin D, including up‐regulation of Fas/FasL, mitochondrial dysfunction, Bcl‐2 family protein modulation, ROS generation, inhibition of inhibitors of apoptosis, induction of mitotic arrest, Mitogen activated protein kinase (MAPK) pathway activation, and suppression of telomerase activity and pro‐survival pathways such as AKT. Platycodin D‐induced apoptosis through multiple mechanisms has been shown in Figure 2.

### Targeting cancer cells by extrinsic apoptosis

The extrinsic apoptotic pathway is triggered by the activation of death receptors (DR) by their respective ligands 1. Theses DRs such as fibroblast‐associated antigen (Fas), tumour necrosis factor receptor‐1, DR3, DR4, DR5 and DR6 are transmembrane receptors containing a cystein‐rich extracellular domain for ligand binding as well as a cytoplasmic domain of about 80 amino acids called death domain which plays a critical role in transmitting the death signals from the cell surface to the intracellular signalling proteins 60, 61, 69. Upon ligand binding, the DRs such as Fas and DR4/5 interact with Fas‐associated death domain (FADD) and procaspase‐8 to form death‐inducing signalling complex (DISC) within the cell. Within this complex, FADD recruits procaspase‐8 through its death effector domain which becomes activated by auto‐proteolysis and dissociates from the DISC to activate apoptotic machinery by directly activating the downstream effector caspase‐3, resulting in cell death *via* the type I extrinsic apoptotic pathway, or cleaves Bid, a pro‐apoptotic member of the Bcl‐2 family, leading to activation of the type II extrinsic apoptotic pathway 1, 70.

Killing cancer by targeting DRs in cancer cells has been considered as a useful and promising approach by researchers 61, 70, 71. Recently, platycodin D has been investigated for its potential value as a source of novel anti‐cancer drug in various cancer cell lines. It has been found to induce extrinsic apoptosis in different human cancer cells including AGS gastric adenocarcinoma, MCF‐7 breast adenocarcinoma, and PC3 prostate cancer and HaCaT cells. In PC3 cells, platycodin D has been shown to induce activation of caspase‐8 45, while in MCF‐7 cells it has been shown to induce activation of caspase‐8 and Bid 72. In HaCaT cells, platycodin D induced extrinsic apoptosis by up‐regulating the expression of Fas and FasL both at mRNA as well as at protein levels and inducing proteolytic cleavage of caspase‐8 and ‐3 in a time‐dependent manner 57. The induction of Fas and FasL appears to come from platycodin D‐induced NF‐κB activation in HaCaT cells. Pre‐treatment of cells with 100 μM TPCK (NF‐κB inhibitor) inhibited the translocation of NF‐κB into nucleus and abrogated platycodin D‐induced apoptosis in HaCaT cells indicating the potential involvement of NF‐κB in platycodin D‐induced apoptosis. Further results from this study revealed that platycodin D induces activation of NF‐κB by stimulating IκB kinase (IKK) activity and IκBα degradation and this inducible NF‐κB is made up of p50 and p65 sub‐units as evident by the reduction in NF‐κB DNA binding activity in the presence of anti‐p50 and anti‐p65 antibodies.

To further confirm the functional link between NF‐κB activation and Fas receptor up‐regulation by platycodin D, HaCaT cells were transfected with luciferase construct pFLF1 containing a promoter region between −1435 and +236 of the Fas receptor gene with the NF‐κB binding site for 18 hrs, followed by a dose‐dependent treatment of platycodin D (10–50 μM) for 3 hrs and luciferase activity was determined. Platycodin D increased luciferase activity dose‐dependently indicating that NF‐κB is involved in positive regulation of Fas receptor promoter.

In AGS gastric cancer cells, platycodin D has been reported to induce type I as well as type II extrinsic apoptosis by inducing the expression of FasL and proteolytic cleavage of caspase‐8 Bid, caspase‐3, caspase‐9 and poly (ADP‐ribose) polymerase (PARP) in a dose‐ and time‐dependent manner 73, 74. Type II extrinsic apoptosis was mediated by translocation of tBid into mitochondrial membrane thereby modulating the expressions of Bcl‐2 family proteins and ultimately releasing cytochrome c into cytosol which is a hallmark of mitochondrial apoptosis. Both type I and type II extrinsic pathways converge at caspase‐3, which is the main executioner of apoptotic cell death 74.

These effects were mediated by platycodin D‐induced activation of p38/AP‐1 (activator protein 1) signalling. Pre‐treatment of cells with SB203580 (p38 inhibitor) completely reversed platycodin D‐induced apoptosis and reduced the up‐regulation of FasL and Bax/Bcl‐2 ratio and suppressed the activation of AP‐1, caspase‐8, ‐9, ‐3 and PARP. These effects were further confirmed by knockdown of p38 expression through siRNA. Moreover, pre‐treatment of cells with parthenolide (AP‐1 inhibitor) inhibited the expressions of pro‐apoptotic proteins Bim and c‐Jun in response to platycodin D. Parthenolide‐mediated inhibition of AP‐1 activation was also verified by EMSA. In addition to p38, platycodin D also increased the phosphorylation of JNK and decreased the phosphorylation of ERK in AGS cells. Platycodin D‐induced apoptosis was not affected by SP600125 (JNK inhibitor), however, U0126 (ERK inhibitor) potentiated the platycodin D‐mediated apoptosis indicating anti‐apoptotic effect of ERK activation in AGS cells. Taken together, these sets of data provided clear evidence that platycodin D induces apoptosis in AGS mainly through p38/AP‐1 signallings 74.

Contrary to the NF‐κB activation in HaCaT cells 57, platycodin D inhibited the activation of NF‐κB in AGS cells 74. NF‐κB activation in HaCaT cells in response to platycodin D treatment appears as apoptosis promoter while platycodin D‐induced apoptosis in AGS cells seems to occur independent of NF‐κB activation. A similar NF‐κB inhibitory effect of platycodin D has been reported in MDA‐MB‐231 breast cancer cells 75 and LPS‐stimulated macrophages 41. This indicates that platycodin D modulates the expression of NF‐κB depending upon the type of cells. Based on available data, it can be concluded that platycodin D inhibits NF‐κB activation in cancer cells while induces NF‐κB expression in normal cells.

### Targeting cancer cells by intrinsic apoptosis

Unlike extrinsic apoptosis, the intrinsic apoptotic pathway is initiated by a diverse array of non‐receptor mediated stimuli that produce intracellular signals that act directly on the target within the cell 60. All of these stimuli alter the functioning of mitochondria. Mitochondria being an important component of the apoptosis execution machinery have been extensively reviewed 1, 61. As the activation of mitochondria has been considered as ‘point of no return’ in apoptotic process, the manipulation of mitochondria activation to initiate apoptotic cell death has been considered as a potential therapeutic approach.

Bcl‐2 family proteins have been envisaged the main mediators of mitochondrial apoptosis 76, 77, 78. Modulation of Bcl‐2 family proteins results in opening of the mitochondrial permeability transition pore, dissipation of mitochondrial membrane potential and subsequent release of many pro‐apoptotic proteins such as cytochrome c, second mitochondrial activator of caspases (Smac/DIABLO), apoptosis inducing factor (AIF), Endonuclease G (Endo G) and caspase‐activated DNase (CAD) from mitochondrial inter‐membrane space into cytosol 79. Cytochrome c, once released into the cytosol, interacts with apoptotic protease activating factor‐1, leading to the activation of caspase‐9 80, 81. Active caspase‐9 then activates caspase‐3, which in turn cleaves substrates such as inhibitor of caspase‐activated DNase (ICAD) and PARP, leading to nucelosomal DNA fragmentation and thus apoptosis. Smac/DIABLO promotes caspases activation through neutralizing the inhibitory effects of inhibitor of apoptosis proteins such as xiap and survivin 82, 83, while AIF and Endo G induce caspase‐independent apoptosis by directly inducing DNA damage and chromatin condensation reffered as stage 1 condensation. ICAD is then released from mitochondria and translocated into nucleus where, after cleavage by caspase‐3, it induces oligonucleosomal DNA fragmentation and more pronounced and advanced chromatin condensation termed as stage II condensation 60, 79.

The apoptotic effects of platycodin D has been investigated in various cancer cell lines. It has been shown to trigger intrinsic apoptosis mainly by modulating the expressions of Bcl‐2 family proteins in different cancer cell lines of human origin including gastric cancer 73, 74, breast cancer 72, liver cancer 56, brain cancer 84 and prostate cancer 45. Platycodin D has significantly reduced the Bcl‐2/Bax ratio and increased the expression of cleaved caspase‐9, caspase‐3 and PARP in PC‐3, AGS, HepG2 and MCF‐7 cancer cells. In U251 human glioma cells, it has been reported to reduce Bcl‐2/Bax ratio and increase DNA fragmentation and caspase‐3 activation. In addition to suppressing Bcl‐2/Bax ratio, platycodin D has been reported to induce the expression of Bim and c‐Jun through AP‐1 transcriptional activity in AGS gastric cancer cells 74. The anti‐cancer activity of platycodin D has also been validated in PC‐3 prostate cancer xenograft model 45. The data demonstrated that 2.5 mg/kg dose of platycodin D suppressed about 56% growth over a period of 4 weeks.

Based on available data, it appears that platycodin D holds the promise to induce intrinsic apoptosis by interacting with Bcl‐2 family proteins. As Bcl‐2 family proteins appear to be an attractive drug target for cancer therapy, it is therefore imperative to explore platycodin D for its potential to target Bcl‐2 family protein in a wide variety of cancer cells both *in vitro* and *in vivo* cancer models to develop it into a promising chemotherapeutic drug.

### Targeting cancer cells by ROS‐mediated apoptosis

Reactive oxygen species are oxygen containing reactive chemical entities which have been shown to play a vital role in various cellular processes including proliferation, cell survival, differentiation, gene expression, and regulating the activity of enzymes and eliminating pathogens and foreign particles 85, 86. Compelling evidence suggests that cancer cells exhibit high oxidative stress which plays important role in cancer cell survival, proliferation, disruption of cell death signalling, angiogenesis, metastasis and drug resistance 87, 88, 89. Recent studies have shown that in contrast to tumour promoting effect of increased ROS, this biochemical property of cancer cells can be exploited for therapeutic benefits. Exploiting the vulnerability of cancer cells with intrinsic oxidative stress to further ROS insult above a toxic threshold level by exogenous ROS generating phytochemicals to selectively kill cancer cells have been shown to be feasible in various *in vitro* and *in vivo* experimental models 82, 87, 88, 89, 90, 91.

Platycodin D has recently been reported to induce apoptosis in U937 leukaemia 92 and MCF‐7 breast cancer cells 72, 93 by targeting ROS metabolism through different mechanisms. In U937 human leukaemia cells, platycodin D (15 μM) have been shown to induce ROS‐dependent apoptosis. Treatment of cells with platycodin D (15 μM) for 48 hrs has induced apoptosis *via* mitochondrial membrane potential dissipation, and increased expression of transcription factor early growth response‐1 (Egr‐1), non‐steroidal anti‐inflammatory drug (NSAID)‐activated gene‐1 (NAG‐1), cleaved caspase‐3 and PARP and DNA fragmentation. Pre‐treatment of cells with NAC (ROS inhibitor) completely reversed while broad‐spectrum caspase inhibitor (z‐VAD‐fmk) partially inhibited the apoptotic effects of platycodin D in U937 cells 92. As platycodin D treatment started to increase ROS generation within 30 min. while induced the expression of EGR‐1 and NAG‐1 after 1 and 2 hrs respectively, it could be concluded that ROS act as an upstream signalling molecule to induce NAG‐1 expression through EGR‐1 activation.

In MCF‐7 breast cancer cells, the apoptotic effect of platycodin D has been found to be associated with ROS generation, mitochondrial dysfunction, Bcl‐2 family protein modulation, caspase‐9, ‐8, ‐3 and PARP cleavage, stress‐activated MAPK proteins activation and ER stress. A reversal of all these ROS‐mediated events was observed with supplementation of ROS scavenger NAC. Pre‐treatment of cells with SP600125 (JNK inhibitor) and SB203580 (p38 inhibitor) has partially reversed the apoptosis indicating that involvement of pathways other than MAPK 72. Platycodin D‐mediated apoptosis *via* ROS signalling has been shown in Figure 2. Further detailed investigations demonstrated that platycodin D induced activation of ASK‐1 by down‐regulating the expression of thioredoxin and phosphorylating ASK‐1 at Threonine 845 and dephosphorylating at Serine 967. Activated ASK‐1 leads to the activation (phosphorylation) of p38 and JNK. Moreover, platycodin D exerted ER stress in MCF‐7 cells by phosphorylating PERK (PKR‐like endoplasmic reticulum kinase) and eukaryotic initiation factor 2 α and glucose‐regulated protein 78/binding immunoglobulin protein and CHOP/GADD153 up‐regulation, and caspase‐4 activation. The induction of ER stress‐related signals by platycodin D treatment was decreased by the antioxidant NAC, suggesting that ROS generation by platycodin D is upstream of ER stress 93.

### Targeting cancer cells by cell cycle‐mediated apoptosis

Cell cycle arrest at a particular checkpoint is one of the major causes of cell death in cancer cells 94. The accurately orchestrated sequence of events that lead to cell division is regulated by a coordinated interaction of a variety of cyclins with their respective cyclin‐dependent kinases at various checkpoints 94, 95. Natural compounds such as taxol and vinca alkaloid that arrest cell cycle at G2/M checkpoint by targeting microtubules have proven to be one of the best classes of cancer chemotherapeutic drugs in both haematopoietic and solid tumours 96, 97. Microtubules represent the best cancer target identified so far 98, 99. Platycodin D has been shown to arrest cell cycle at G2/M checkpoint in various cancer cell lines. In human hepatoma HepG2 cells, it induced G2/M arrest in a dose‐dependent manner 100. In U937 leukaemia cells, platycodin D appears to arrest cell cycle at G2 phase by inhibition of cyclin B1/cdk1 complex *via* Wee1 expression and M phase by promoting tubulin polymerization 101. Taken together, the data suggest that platycodin D induced endoreduplication (END) through mitotic arrest as evident by increased expression of cdk2 and mitosis markers such as histone H3 serine 10 phosphorylation, BubR1 and MPM‐2 and this END ultimately leads to mitochondrial apoptosis. Unlike eteposide and merbarone which induce END *via* Topo II inhibition, platycodin‐induced END is not associated with Topo II activity.

**Figure 3:**
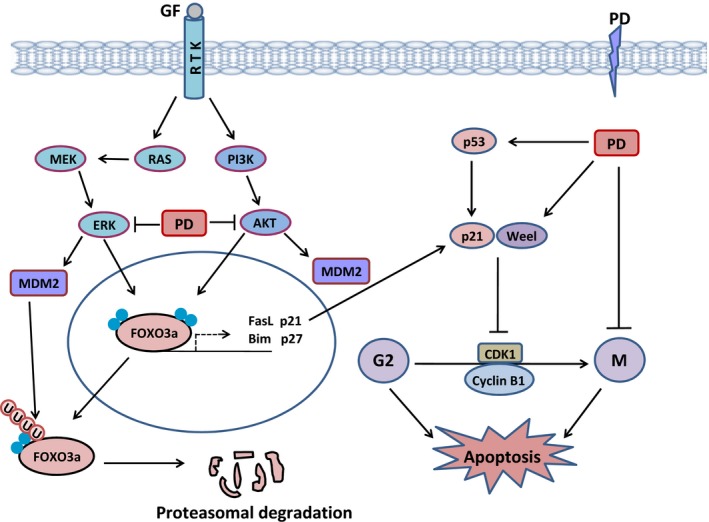
A schematic representation of platycodin D‐induced G2/M phase cell cycle arrest in cancer cells. Platycodin D inhibits G2‐M phase transition by increasing the expression of transcription factor FOXO3a, p53, p21 and Weel, and decreasing the expression of MDM2 and induces M phase arrest by sustainable tubulin polymerization. Binding of ligand such as growth factor (EGF) to the growth factor receptor (EGFR) promotes the activation of downstream pro‐survival signalling pathways such as PI3K/AKT and MAPK/ERK pathways. Activated AKT and ERK translocate to the nucleus and inhibit the transcription of apoptosis promoting (Bim, FasL, TRAIL) and cell cycle regulatory genes (p21, p27) by phosphorylating FOXO3a and exporting it out of nucleus. ERK regulates the expression of MDM2 that ubiquitinates FOXO3a in response to ERK pathway activation leading to proteasomal degradation. Platycodin D induces G2/M phase arrest and apoptosis by increasing the expression of FOXO3a in nucleus by inhibiting AKT and ERK.

Platycodin D has also been found to induce G2/M phase arrest in PC3 cells in a dose‐dependent manner 45. It has been shown to inhibit the expressions of E2F1, MDM2, Cyclins (B1 & D1) and CDKs (1, 2, 4 & 6). These effects were associated with up‐regulation of tumour suppressor protein FOXO3a (Forkhead box transcription factor) and its direct downstream target genes p21 and p27 in PC3 cells. Platycodin D‐induced expression of FOXO3a appears to come from suppressive effects of platycodin D on PI3K/AKT and ERK pathways as shown in Figure 3.

### Targeting cancer cells by non‐specified apoptosis

In addition to aforementioned apoptotic cell death processes which can be initiated either by activation and ligation of DRs to their respective ligands or by mitochondrial activation, platycodin D has been shown to induce apoptotic cell death in U937 leukaemia cells by inducing DNA fragmentation, LDH release and cleavage of caspase‐3 and PARP in a dose‐dependent manner through a non‐specified apoptotic process 102. The apoptotic cell death found to be linked with reduced activity of telomerase *via* down‐regulation of hTERT (Telomerase reverse transcriptase) expression. Platycodin D treatment decreased the protein levels of c‐Myc and Sp1 (Regulators of hTERT transcription) and reduced their DNA binding activity. In addition to hTERT transcription inhibition, platycodin D treatment also suppressed the activity of telomerase by inhibiting the phosphorylation and nuclear translocation of hTERT *via* inhibition of AKT activation. The set of data demonstrate that cytotoxic effects of platycodin D in U937 leukaemia cells seems to result from suppression of telomerase activity through transcriptional and post‐transcriptional inhibition of hTERT activity 102. Platycodin D has also been shown to inhibit growth and DNA synthesis and promote apoptosis and caspase‐3 activity in MDA‐MB‐231 cells in a dose‐dependent manner 103.

## Targeting autophagy in cancer cells

Autophagy is an evolutionary highly conserved catabolic process that plays a vital role in degradation of misfolded proteins and damaged organelles 104, 105. It plays a very prominent role in various physiological and pathological conditions such as cancer 104, 106. A substantial amount of research evidence exists to support autophagy as a bonafide tumour suppressor mechanism. Autophagic cell death or type II programmed cell death is an alternative mechanism of cancer cell death in apoptosis deficient/resistant cells. Accumulating evidence from various studies has shown that natural compounds induce autophagic cell death in apoptosis defective cells 106, 107, 108. The connection between apoptosis and autophagy is complex and is currently being investigated for therapeutic benefits 107. For example, anti‐apoptotic Bcl‐2 protein also inhibits autophagy by inhibiting beclin; an autophagy gene initially identified as tumour suppressor. Inhibition of anti‐apoptotic Bcl‐2 protein has been shown to induce apoptosis as well as autophagy in cancer cells 108. Inhibition of protective autophagy has been shown to induce apoptosis and increase the therapeutic efficacy of cisplatin and 5‐fluorouracil in human cancers. Other studies showed that autophagy switches to apoptosis and inhibition of autophagy delays apoptosis 106. Thus, identification and characterization of the type of autophagy and interaction between apoptosis and autophagy may provide a unique strategy for the development of highly selective and more effective anti‐cancer drugs for various carcinomas.

LC3 is a hallmark of autophagy and conversion of LC3‐I to LC3‐II shows autophagy induction 109, 110. Recently, platycodin D has been explored for its potential to induce autophagy. The compound was found to be potent inducer of autophagy at a concentration range of 1.25–30 μM in a wide range of cancer cells including HepG2, Hep3B, MCF‐7, MDA‐MB‐231, A549, 95D and NCI‐H460 cells 111. In Hep3B, MCF‐7, MDA‐MB‐231, A549 and 95D cells, platycodin D triggered autophagy in a dose‐ and time‐dependent manner by increasing the expression of LC3‐II. In HepG2 cells, an increased level of LC3‐II expression was observed when cells were treated with a combination of platycodin D and late‐stage autophagy inhibitors (CQ and BAF) compared to platycodin D treatment alone. Combine treatment of cells with platycodin D and autophagy inhibitors enhanced the apoptotic effect in HepG2 cells indicating that platycodin D induced protective autophagy in HepG2 cells. Moreover, platycodin D increased phosphorylation of ERK and U0126 (MEK inhibitor) reduced the phosphorylation of ERK and attenuated platycodin D‐induced expression of LC3‐II indicating that ERK activation is involved in platycodin D‐mediated autophagy in HepG2 cells 111.

**Figure 4:**
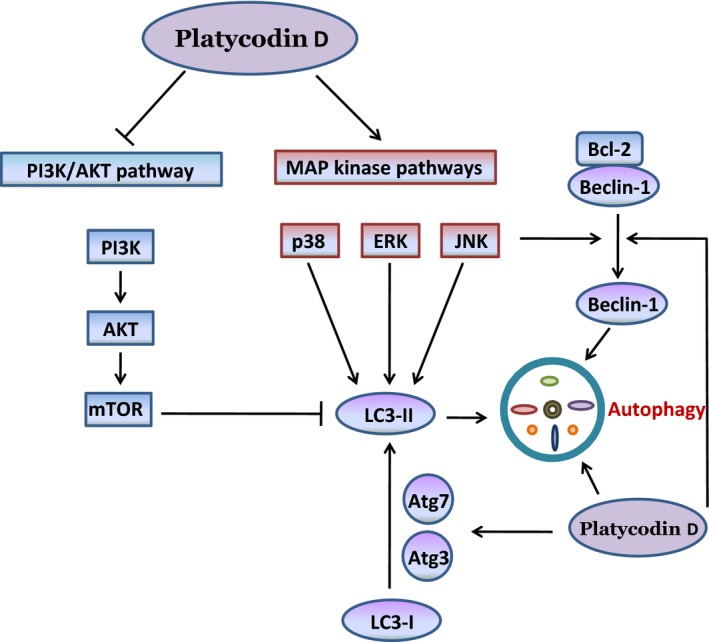
Molecular mechanism of platycodin D‐induced autophagy in cancer cells: Platycodin D induces autophagy through PI3K/AKT and MAPK pathways. Platycodin D converted LC3‐I to LC3‐II by increasing the expression of Beclin‐1, Atg3 and Atg 7. Inhibition of PI3K/AKT and activation of MAPK signalling pathways by platycodin D resulted in autophagy by accumulating LC3‐II in cancer cells (⊢Inhibition; ⟵ Activation).

In A549 and NCI‐H460 human non‐small cell lung carcinoma cells, platycodin D induced autophagy by increasing the conversion of LC3‐I to LC3‐II and accumulation of Atg‐3, Atg‐7 and Beclin in a dose‐ and time‐dependent manner. Platycodin D treatment reduced the phosphorylation of several members of the PI3K/Akt/mTOR signalling pathway such as p‐Akt (Ser473), p‐p70S6K (Thr389) and p‐4EBP1 (Thr37/46), and increased the expression of phosphorylated p38 and JNK in A549 and NCI‐H460 cells 112. Platycodin D co‐treatment with LY294002 (PI3K inhibitor) or rapamycin increased the expression of LC3‐II in a synergistic fashion. Moreover, platycodin D reversed insulin‐mediated activation of PI3K/Akt/mTOR signalling and suppression of autophagy in these cancer cell lines. Platycodin D also increased the phosphorylation of p38 MAPK and JNK. Pre‐treatment of cells with SB203580 (p38 inhibitor) and SP600125 (JNK inhibitor) decreased the ratio of LC3‐II/LC3‐I as compared to platycodin D treatment alone. Taken together, platycodin D trigger autophagy *via* inhibiting PI3K/Akt/mTOR signalling pathway and activating JNK and p38MAPK signalling pathway in A549 and NCI‐H460 cells. The effect of platycodin D on autophagy induction has been represented in Figure 4.

## Targeting tumour cell development and progression with platycodin D

Apart from type I and type II programmed cell death, platycodin D has been shown to interfere with tumour progression by interacting with multiple targets.

### Targeting proliferation of cancer cells

In normal tissues, cell proliferation is controlled by irreversible entry into post‐mitotic terminal differentiation 113. Cancer cells escape this terminal differentiation by up‐regulating the expression of c‐Myc which is a potent inhibitor of differentiation in many cell lineages 113, 114. Myc has been found deregulated in nearly half of the human solid tumours and leukaemia and appears frequently associated with cancer progression 115. Besides c‐Myc, overexpression of cylin D1 116 and phospholipase Cγ1 (PLCγ1) 117 has been linked with higher rates of cellular proliferation in many cancers. Platycodin D inhibited proliferation in several human cancer cells in a time‐ and dose‐dependent manner 41, 45, 56, 72, 73, 74, 75, 84, 92, 100, 101, 102, 118. Apart from its anti‐proliferative effects determined by MTT assay, platycodin D has been shown to inhibit the expressions of c‐Myc in U937 leukaemia cells 102, phosphorylated PLCγ1 in HUVECs 118 and cyclin D1 in PC3 prostate 45 and AGS gastric 73 cancer cells. Platycodin D also inhibited tumour growth and expression of Ki‐67, a well known marker of cell proliferation in MDA‐MB‐231 xenograft tumours in athymic nude mice at a dose of 5 mg/kg 75.

### Targeting pro‐survival pathways in cancer cells

One of the major characteristic features of cancer cells is their ability to avoid apoptotic cell death by activating pro‐survival signalling. PI3K/AKT 1 and NF‐κB 119, 120 signalling pathways are frequently overexpressed in a variety of human cancers. Several anti‐apoptotic proteins such as Bcl‐2 and survivin which are known to be crucial for cancer cell survival are the downstream target genes of AKT and NF‐κB. In MDA‐MB‐231 breast cancer cells, platycodin D inhibits both constitutive and EGF‐induced activation of AKT and mTOR 75. It also inhibits constitutive and LPS‐stimulated activation of NF‐κB in MDA‐MB‐231 75 and RAW 264.7 41 cells respectively. Platycodin D has also been reported to inhibit PI3K/AKT signalling pathway in HUVECs 118, HepG2 100, U251 84, NCI‐H460, A549 112, U937 102 and AGS 73 cells.

In addition to AKT and NF‐κB signalling, many studies have demonstrated that platycodin D is a potent inhibitor of anti‐apoptotic protein Bcl‐2 and survivin. It down‐regulated Bcl‐2 in MCF‐7 72, AGS 73, HepG2 100, PC3 45 and U251 84, and survivin in HepG2 56 and AGS 73 cells.

### Targeting angiogenesis in cancer cells

Angiogenesis is a complex physiological process by which new blood vessels are formed from pre‐existing structures 121. Angiogenesis plays a vital role in the growth and survival of solid neoplasm and is considered as a hallmark of cancer progression. Although angiogenesis is regulated by multiple signalling pathways, it is now universally accepted that VEGF and its cognate receptor VEGFR‐2 are the most prominent regulators 122. VEGF signalling stimulates cellular pathways that promote rapid tumour growth and facilitate metastatic potential of cancer by forming new blood vessels for adequate blood supply 122. The development of highly selective drugs that target VEGF such as Bevacizumab or VEGFR‐2 such as Sorafenib has improved the prognosis of patients with metastatic cancer but fail to achieve the desire outcome because of activation of other compensatory mechanisms such as fibroblast growth factor and COX‐2/PGE2. Thus, simultaneous targeting of VEGF and other compensatory pathways may provide a useful strategy for the development of most effective drugs for metastatic tumours.

Platycodin D has recently been investigated for its anti‐angiogenic effects. Luan *et al*. 118 showed that platycodin D inhibits the growth, migration and tube formation in HUVEC in a dose‐dependent manner at a very low concentration (0.3–30 μM). It also inhibits angiogenesis in chick embryo chorioallantoic membrane. The anti‐angiogenic effect of platycodin D in tumour was validated using HCT‐15 xenograft mouse model. Platycodin D inhibited tumour growth, decreased tumour microvessel density and increased apoptosis in tumour tissue at a dose of 6 mg/kg/day for 14 days without any toxic effects to mice. The anti‐angiogenic activity of platycodin D appeared to result from its suppressive effect on VEGF‐induced VEGFR‐2 phosphorylation and its downstream targets such as AKT, JAK‐2, Src and PLCγ1 118. Platycodin D has decreased the expression of pro‐angiogenic factor VEGF and COX‐2 in a dose‐dependent manner (10–20 μM) in AGS cells 73. In RAW 264.7 cells, platycodin D down‐regulates the LPS‐induced expression of COX‐2 and iNOS at both transcriptional and translational levels. Platycodin D also inhibited the LPS‐induced synthesis of prostaglandin E2 (PGE2) the enzymatic product of COX‐2 in RAW 264.7 cells 41. Collectively, platycodin D exerts angio‐suppressive activity by regulating the expressions of VEGF, VEGFR‐2, iNOS, COX‐2 and PGE2 in different cells.

### Targeting invasion and metastasis

Tumour invasion and metastasis are complex, multi‐step biochemical processes and are considered as one of the hallmarks of cancer biology. Invasion is the direct migration and penetration of cancer cells into neighbouring tissues while metastasis is the ability of cancer cells to penetrate into lymphatic or blood vessels, circulate through the bloodstream and then invade in normal tissues elsewhere in the body 123. Degradation of basement membrane and remodelling of extracellular matrix (ECM) are the characteristic features of cancer invasion and metastasis. Matrix metalloproteinases (MMPs) especially MMP‐2 and ‐9 are aberrantly expressed in cancers and are believed to play vital role in cancer invasion and metastasis 124, 125. Metastasis has been reported to responsible for about 90% of cancer patient deaths and is considered as the most challenging obstacle in successful treatment of cancer 125.

Platycodin D has been shown to inhibit migration and invasion of MDA‐MB‐231 cells in a dose‐dependent manner (5–15 μM) as seen from wound healing assay and matrigel transwell chamber assay respectively. Treatment of cells with 15 μM platycodin D inhibited the adhesion of cells to fibronectin, collagen type I, laminin and fibrinogen by 42%, 54%, 49% and 50% respectively indicating that suppressive effect of the compound on MDA‐MB‐231 cells invasion is associated with decreased adhesion of cells to ECM proteins. Zymographic analysis revealed that platycodin D markedly inhibited the activity of MMP‐9, while MMP‐2 activity was slightly decreased. Platycodin D also down‐regulates the mRNA expression of MMP‐9 as evident from real‐time PCR analysis 75. Platycodin D's anti‐invasion and anti‐metastatic activity may be associated to its ability to inhibit proteolytic activity and mRNA expression of MMP‐9.

In HepG2 cells, platycodin D inhibited colony formation and suppressed adhesion to matrigel dose‐dependently (1.25–10 μM). Platycodin D also inhibits 12‐O‐tetradecanoylphorbol 13‐acetate (TPA)‐induced migration and invasion of HepG2 cells as seen from transwell chamber assay 56. Platycodin D has also decreased the expression and phosphorylation of FAK, a component of cell substratum adhesion, in AGS 74 and HUVEC 118. Among other players of invasion and metastasis, platycodin D reduced VEGF‐induced phosphorylation of JAK2 and Src in human umbilical vein endothelial cells in a dose‐dependent fashion 118.

## *In vivo* anti‐tumour effects of platycodin D

Apart from *in vitro* anti‐cancer activity of platycodin D, various tumour models and drug administration routes have been tested for platycodin D *in vivo*. Both intraperitoneal as well as oral administration of platycodin D has been shown to exhibit significant anti‐tumour activity. Intraperitoneal administration of platycodin D at a dose of 5 mg/kg bodyweight significantly reduced the volume and size of MDA‐MB‐231 xenograft tumour. Immunohistochemical analysis of tumour tissues shows that platycodin D inhibited the expression of Ki‐67 and EGFR 75. EGFR inhibition seems to be major mechanism of platycod in D's *in vivo* anti‐tumour efficacy as demonstrated by *in vitro* cell studies.

In PC3 prostate cancer xenograft model, intraperitoneal administration of 2.5 mg/kg platycodin D achieved 56% tumour growth inhibition. At a very low dose of 1 mg/kg, platycodin D also reduced tumour size but not significantly. Immunobloting analysis of tumour tissues showed that platycodin D enhanced the expression of FOXO3a, p21, p27, Bax and cleaved PARP, while decreased the expression of MDM2, Bcl‐2, cyclin B1 and D1 and CDK‐1, ‐2, ‐4 and ‐6. FOXO3a seems to hold the promise of platycodin D‐mediated anti‐cancer activity 45.

A recent study by Park *et al*. 44 has shown anti‐tumour effect of platycodin D in H520 cell xenograft Balb/c *nu‐nu* nude mice. Oral administration of 50, 100 and 200 mg/kg platycodin D, once a day for 5 weeks from 15 days after tumour cells inoculation has significantly reduced the tumour size and weight with remarkable immune‐stimulatory and anti‐cachexia effects in a dose‐dependent manner. Gemcitabine (160 mg/kg) has been used as positive control in this study. Gemcitabine also exerted potent anti‐tumour effects but aggravated the cancer‐related immune suppressions and cachexia in this experiment.

More recently, Lee *et al*. 103 has shown that platycodin D inhibits breast cancer‐induced bone destruction by inhibiting osteoclastogenesis and inducing apoptosis in MDA‐MB‐231 breast cancer cells in Balb/c *nu/nu* mice. Oral administration of 2 mg/kg platycodin D has significantly inhibited MDA‐MB‐231 cell‐induced osteolysis and tumour growth in bone marrow in intratibial mouse model. Collective data of this study demonstrate that platycodin D inhibits recombinant mouse receptor activator of nuclear factor kappa‐B ligand‐induced osteoclast formation by inhibiting the expression and nuclear translocation of NFATc1 and c‐Fos in bone marrow‐derived macrophages and ultimately reduces osteoclast‐mediated bone resorption.

In addition to anti‐tumour effects, platycodin D has also been explored for its toxic effects on general body organ using male and female ICR mice. A single oral dose of platycodin D up to 2000 mg/kg could be safely administered without causing treatment related mortality, abnormal clinical signs, remarkable changes in body and organ weights and significant histopathological changes in 14 principal organs till 14 days of post‐treatment in both male and female mice 43. The Korean Food and Drug Administration 43 guidelines indicate that the maximum platycodin D dosage recommendation is 2000 mg/kg.

Platycodin D has also been demonstrated to ameliorate cisplatin‐induced nephrotoxicity in ICR mice by attenuating oxidative stress and cell death in kidney 58. It should also be noted that platycodin D was well tolerated and no mortality or any sign of pharmacotoxicity was observed in aforementioned tumour bearing nude mice during all experimental periods.

## Platycodin D in combination therapy

The combined application of saponins with other anti‐tumour drugs has drawn a considerable attention in cancer chemotherapy because of huge structural diversity and adjuvant activity of saponins. Because of excellent anti‐tumour effect of platycodin D in various *in vitro* and *in vivo* cancer models, it has recently been investigated for its combined application with other anti‐tumour drugs in various cell lines of breast cancer. In MCF‐7 (oestrogen receptor positive) and MDA‐MB‐231 (oestrogen receptor negative) cell lines of breast cancer, platycodin D has been shown to enhance the anti‐cancer activity of doxorubicin. While treatment with either platycodin D (10 μM) or Doxorubicin (2.5 μM) *in vitro* resulted in apoptotic cell death, a combine treatment of both demonstrated a significant growth suppressive effect and PARP cleavage in both cell lines compared to treatment with either agent alone 126. As platycodin D increased the intracellular accumulation of doxorubicin in given concentration only in MDA‐MB‐231 cells, the equally increased growth suppressive effects of combine treatment in both cell lines could not be associated with enhanced accumulation of doxorubicin in cells.

Platycodin D has also been shown to exhibit greater growth inhibitory and anti‐invasive effects in combination with osthol in MDA‐MB‐231 and 4T1 breast carcinoma cells 42. Platycodin D (75 μM) in combination with osthol (15 μM) synergistically inhibits the growth and invasion of both cancer cell lines. Both these compounds have been shown to exert varying effects on the expression of TβRII, Smad2 and Smad3; however, a combine treatment of both agents effectively reduced the mRNA and protein expressions of TβRII, Smad2 and Smad3 in both cell lines. Moreover, combine treatment effectively decreased the TGF (transforming growth factor)‐β‐induced phosphorylation of Smad2 and Smad3. Taken together, the data demonstrate that platycodin D‐osthol combination inhibits proliferation and blocks invasion of MDA‐MB‐231 and 4T1 breast cancer cells by inhibiting TGF‐β/Smad signalling. Thus, platycodin D may be developed into a potential lead compound for treating breast cancer in combination with other chemotherapeutic agents.

## Concluding remarks and future perspectives

In this review, we have highlighted the recent progress of platycodin D in various *in vitro* and *in vivo* cancer models. Collective data from multitudinous studies has provided persuasive evidence for promising role of platycodin D in treatment of many type of cancers. It has been shown to interact with multiple mechanisms to kill cancer cells in a wide variety of tumours. The most exciting possibility to develop platycodin D into a lead is its low toxicity to normal cells and good adjuvant property. The major drawback to develop saponins into a successful chemotherapeutic drug is their severe toxicity to normal cells. Contrary to most of the saponins, platycodin D has been shown to exert less toxic effects on normal cells; however, the exact mechanism still remains unexplored. As platycodin D has been shown to exhibit excellent anti‐cancer activity by interfering with multiple mechanisms which are the hallmark of cancer, further detailed studies regarding its structure–activity relationship for haemolysis and adjuvant property should be conducted with special focus on the role of sugar chains. As evident by study of Chun *et al*.^73^, deglycosylation of platycodin D at C‐3 and C‐28 reduces its cytotoxicity while *o*‐acetylation at rahmnose C‐2 or C‐3 as well as dehydroxylation at C‐24 of platycodin D has been shown to increase its cytotoxicity against various cancer cells. Medicinal chemistry studies may be needed to improve the anti‐cancer and adjuvant activity and reduce the haemolytic ability of platycodin D to develop it into a successful chemotherapeutic drug either alone or in synergistic combination with other drugs.

It is hoped that the data collected here will provide the researchers with valuable information and establish the basis for the future development of platycodin D into a promising anti‐cancer drug in the years to come.

## Conflicts of interest

The authors confirm that there are no conflicts of interest.

## References

[1] Khan M , Maryam A , Qazi JI , *et al* Targeting apoptosis and multiple signaling pathways with icariside II in cancer cells. Int J Biol Sci. 2015; 11: 1100–12. 26221076 10.7150/ijbs.11595 PMC4515820

[2] Ferlay J , Soerjomataram I , Dikshit R , *et al* Cancer incidence and mortality worldwide: sources, methods and major patterns in GLOBOCAN 2012. Int J Cancer. 2015; 136: E359–86. 25220842 10.1002/ijc.29210

[3] Begnini KR , Moura de Leon PM , Thurow H , *et al* Brazilian red propolis induces apoptosis‐like cell death and decreases migration potential in bladder cancer cells. Evid Based Complement Alternat Med. 2014; 2014: 639856. 25530785 10.1155/2014/639856 PMC4235187

[4] Millimouno FM , Dong J , Yang L , *et al* Targeting apoptosis pathways in cancer and perspectives with natural compounds from mother nature. Cancer Prev Res. 2014; 7: 1081–107. 10.1158/1940-6207.CAPR-14-0136 25161295

[5] Rahmani AH , Alzohairy MA , Khan MA , *et al* Therapeutic implications of black seed and its constituent thymoquinone in the prevention of cancer through inactivation and activation of molecular pathways. Evid Based Complement Alternat Med. 2014; 2014: 724658. 24959190 10.1155/2014/724658 PMC4052177

[6] Coco S , De Mariano M , Valdora F , *et al* Identification of ALK germline mutation (3605delG) in pediatric anaplastic medulloblastoma. J Hum Genet. 2012; 57: 682–4. 22810114 10.1038/jhg.2012.87

[7] Shin S , Kim J , Yoon SO , *et al* ALK‐positive anaplastic large cell lymphoma with TPM3‐ALK translocation. Leuk Res. 2012; 36: e143–5. 22591683 10.1016/j.leukres.2012.04.008

[8] Holohan C , Van Schaeybroeck S , Longley DB , *et al* Cancer drug resistance: an evolving paradigm. Nat Rev Cancer. 2013; 13: 714–26. 24060863 10.1038/nrc3599

[9] Faivre S , Djelloul S , Raymond E . New paradigms in anticancer therapy: targeting multiple signaling pathways with kinase inhibitors. Semin Oncol. 2006; 33: 407–20. 16890796 10.1053/j.seminoncol.2006.04.005

[10] Shu L , Cheung KL , Khor TO , *et al* Phytochemicals: cancer chemoprevention and suppression of tumor onset and metastasis. Cancer Metastasis Rev. 2010; 29: 483–502. 20798979 10.1007/s10555-010-9239-y

[11] Amin AR , Kucuk O , Khuri FR , *et al* Perspectives for cancer prevention with natural compounds. J Clin Oncol. 2009; 27: 2712–25. 19414669 10.1200/JCO.2008.20.6235 PMC2690394

[12] Weng CJ , Yen GC . Chemopreventive effects of dietary phytochemicals against cancer invasion and metastasis: phenolic acids, monophenol, polyphenol, and their derivatives. Cancer Treat Rev. 2012; 38: 76–87. 21481535 10.1016/j.ctrv.2011.03.001

[13] Cragg GM , Newman DJ . Plants as a source of anti‐cancer agents. J Ethnopharmacol. 2005; 100: 72–9. 16009521 10.1016/j.jep.2005.05.011

[14] Balunas MJ , Kinghorn AD . Drug discovery from medicinal plants. Life Sci. 2005; 78: 431–41. 16198377 10.1016/j.lfs.2005.09.012

[15] Chin YW , Balunas MJ , Chai HB , *et al* Drug discovery from natural sources. AAPS J. 2006; 8: E239–53. 16796374 10.1007/BF02854894 PMC3231566

[16] Dall'Acqua S . Natural products as antimitotic agents. Curr Top Med Chem. 2014; 14: 2272–85. 25434355 10.2174/1568026614666141130095311

[17] Borris RP . Natural products research: perspectives from a major pharmaceutical company. J Ethnopharmacol. 1996; 51: 29–38. 9213624 10.1016/0378-8741(95)01347-4

[18] Juarez P . Plant‐derived anticancer agents: a promising treatment for bone metastasis. BoneKEy Rep. 2014; 3 Doi:10.1038/bonekey.2014.94. 10.1038/bonekey.2014.94 PMC5307970 28243436

[19] Ogbourne SM , Parsons PG . The value of nature's natural product library for the discovery of New Chemical Entities: the discovery of ingenol mebutate. Fitoterapia. 2014; 98: 36–44. 25016953 10.1016/j.fitote.2014.07.002

[20] Newman DJ , Cragg GM . Natural products as sources of new drugs over the last 25 years. J Nat Prod. 2007; 70: 461–77. 17309302 10.1021/np068054v

[21] Jones AM , Chory J , Dangl JL , *et al* The impact of Arabidopsis on human health: diversifying our portfolio. Cell. 2008; 133: 939–43. 18555767 10.1016/j.cell.2008.05.040 PMC3124625

[22] Ji HF , Li XJ , Zhang HY . Natural products and drug discovery. Can thousands of years of ancient medical knowledge lead us to new and powerful drug combinations in the fight against cancer and dementia? EMBO Rep. 2009; 10: 194–200. 19229284 10.1038/embor.2009.12 PMC2658564

[23] Taylor LP , Grotewold E . Flavonoids as developmental regulators. Curr Opin Plant Biol. 2005; 8: 317–23. 15860429 10.1016/j.pbi.2005.03.005

[24] Koch MA , Schuffenhauer A , Scheck M , *et al* Charting biologically relevant chemical space: a structural classification of natural products (SCONP). Proc Natl Acad Sci USA. 2005; 102: 17272–7. 16301544 10.1073/pnas.0503647102 PMC1297657

[25] Monks NR , Li B , Gunjan S , *et al* Natural Products Genomics: a novel approach for the discovery of anti‐cancer therapeutics. J Pharmacol Toxicol Methods. 2011; 64: 217–25. 21539926 10.1016/j.vascn.2011.04.002

[26] Baikar S , Malpathak N . Secondary metabolites as DNA topoisomerase inhibitors: a new era towards designing of anticancer drugs. Pharmacogn Rev. 2010; 4: 12–26. 22228937 10.4103/0973-7847.65320 PMC3249898

[27] Evidente A , Kornienko A , Lefranc F , *et al* Sesterterpenoids with anticancer activity. Curr Med Chem. 2015; 22: 3502–22. 26295461 10.2174/0929867322666150821101047 PMC4955362

[28] Onrubia M , Cusido RM , Ramirez K , *et al* Bioprocessing of plant *in vitro* systems for the mass production of pharmaceutically important metabolites: paclitaxel and its derivatives. Curr Med Chem. 2013; 20: 880–91. 23210777

[29] Stahlhut SG , Siedler S , Malla S , *et al* Assembly of a novel biosynthetic pathway for production of the plant flavonoid fisetin in Escherichia coli. Metab Eng. 2015; 31: 84–93. 26192693 10.1016/j.ymben.2015.07.002

[30] Lecci RM , Logrieco A , Leone A . Pro‐oxidative action of polyphenols as action mechanism for their pro‐apoptotic activity. Anticancer Agents Med Chem. 2014; 14: 1363–75. 25244914 10.2174/1871520614666140922121014

[31] Chang TH , Hsieh FL , Ko TP , *et al* Structure of a heterotetrameric geranyl pyrophosphate synthase from mint (*Mentha piperita*) reveals intersubunit regulation. Plant Cell. 2010; 22: 454–67. 20139160 10.1105/tpc.109.071738 PMC2845413

[32] Tian X , Tang H , Lin H , *et al* Saponins: the potential chemotherapeutic agents in pursuing new anti‐glioblastoma drugs. Mini Rev Med Chem. 2013; 13: 1709–24. 24032516 10.2174/13895575113136660083

[33] Sarkar S , Gopal PK , Paul S . Diterpenoids‐ potential chemopreventive and chemotherapeutic agents in leukemia. Curr Pharm Biotechnol. 2014; 15: 127–42. 24934479 10.2174/1389201015666140604121658

[34] Gach K , Dlugosz A , Janecka A . The role of oxidative stress in anticancer activity of sesquiterpene lactones. Naunyn Schmiedebergs Arch Pharmacol. 2015; 388: 477–86. 25656627 10.1007/s00210-015-1096-3

[35] Man S , Gao W , Zhang Y , *et al* Chemical study and medical application of saponins as anti‐cancer agents. Fitoterapia. 2010; 81: 703–14. 20550961 10.1016/j.fitote.2010.06.004

[36] Xie Y , Ye YP , Sun HX , *et al* Contribution of the glycidic moieties to the haemolytic and adjuvant activity of platycodigenin‐type saponins from the root of *Platycodon grandiflorum* . Vaccine. 2008; 26: 3452–60. 18501482 10.1016/j.vaccine.2008.04.023

[37] Nyakudya E , Jeong JH , Lee NK , *et al* Platycosides from the roots of *Platycodon grandiflorum* and their health benefits. Prev Nutr Food Sci. 2014; 19: 59–68. 25054103 10.3746/pnf.2014.19.2.059 PMC4103729

[38] Fuchs H , Bachran D , Panjideh H , *et al* Saponins as tool for improved targeted tumor therapies. Curr Drug Targets. 2009; 10: 140–51. 19199910 10.2174/138945009787354584

[39] Woldemichael GM , Wink M . Identification and biological activities of triterpenoid saponins from *Chenopodium quinoa* . J Agric Food Chem. 2001; 49: 2327–32. 11368598 10.1021/jf0013499

[40] Sun H , Chen L , Wang J , *et al* Structure‐function relationship of the saponins from the roots of *Platycodon grandiflorum* for hemolytic and adjuvant activity. Int Immunopharmacol. 2011; 11: 2047–56. 21945665 10.1016/j.intimp.2011.08.018

[41] Ahn KS , Noh EJ , Zhao HL , *et al* Inhibition of inducible nitric oxide synthase and cyclooxygenase II by *Platycodon grandiflorum* saponins *via* suppression of nuclear factor‐kappaB activation in RAW 264.7 cells. Life Sci. 2005; 76: 2315–28. 15748625 10.1016/j.lfs.2004.10.042

[42] Ye Y , Han X , Guo B , *et al* Combination treatment with platycodin D and osthole inhibits cell proliferation and invasion in mammary carcinoma cell lines. Environ Toxicol Pharmacol. 2013; 36: 115–24. 23603464 10.1016/j.etap.2013.03.012

[43] Lee WH , Gam CO , Ku SK , *et al* Single oral dose toxicity test of platycodin d, a saponin from platycodin radix in mice. Toxicol Res. 2011; 27: 217–24. 24278575 10.5487/TR.2011.27.4.217 PMC3834385

[44] Park JC , Lee YJ , Choi HY , *et al* *In vivo* and *in vitro* antitumor effects of platycodin d, a saponin purified from platycodi radix on the h520 lung cancer cell. Evid Based Complement Alternat Med. 2014; 2014: 478653. 25477992 10.1155/2014/478653 PMC4247928

[45] Zhou R , Lu Z , Liu K , *et al* Platycodin D induces tumor growth arrest by activating FOXO3a expression in prostate cancer *in vitro* and *in vivo* . Curr Cancer Drug Targets. 2015; 14: 860–71. 25431082 10.2174/1568009614666141128104642 PMC4997962

[46] Jia G , Wang Q , Wang R , *et al* Tubeimoside‐1 induces glioma apoptosis through regulation of Bax/Bcl‐2 and the ROS/Cytochrome C/Caspase‐3 pathway. Onco Targets Ther. 2015; 8: 303–11. 25674005 10.2147/OTT.S76063 PMC4321652

[47] Xu Y , Chiu JF , He QY , *et al* Tubeimoside‐1 exerts cytotoxicity in HeLa cells through mitochondrial dysfunction and endoplasmic reticulum stress pathways. J Proteome Res. 2009; 8: 1585–93. 19215086 10.1021/pr801001j

[48] Yin Y , Chen W , Tang C , *et al* NF‐kappaB, JNK and p53 pathways are involved in tubeimoside‐1‐induced apoptosis in HepG2 cells with oxidative stress and G(2)/M cell cycle arrest. Food Chem Toxicol. 2011; 49: 3046–54. 22005259 10.1016/j.fct.2011.10.001

[49] Huang P , Yu C , Liu XQ , *et al* Cytotoxicity of tubeimoside I in human choriocarcinoma JEG‐3 cells by induction of cytochrome c release and apoptosis *via* the mitochondrial‐related signaling pathway. Int J Mol Med. 2011; 28: 579–87. 21687933 10.3892/ijmm.2011.727

[50] Ma R , Song G , You W , *et al* Anti‐microtubule activity of tubeimoside I and its colchicine binding site of tubulin. Cancer Chemother Pharmacol. 2008; 62: 559–68. 18030471 10.1007/s00280-007-0635-0 PMC2493533

[51] Xu Y , Wang G , Chen Q , *et al* Intrinsic apoptotic pathway and G2/M cell cycle arrest involved in tubeimoside I‐induced EC109 cell death. Chin J Cancer Res. 2013; 25: 312–21. 23825908 10.3978/j.issn.1000-9604.2013.06.03 PMC3696712

[52] Tada A , Kaneiwa Y , Shoji J , *et al* Studies on the saponins of the root of *Platycodon grandiflorum* A. De Candolle. I. Isolation and the structure of platycodin‐D. Chem Pharm Bull (Tokyo). 1975; 23: 2965–72. 1218442 10.1248/cpb.23.2965

[53] Li W , Liu Y , Wang Z , *et al* Platycodin D isolated from the aerial parts of *Platycodon grandiflorum* protects alcohol‐induced liver injury in mice. Food Funct. 2015; 6: 1418–27. 25927324 10.1039/c5fo00094g

[54] Zhang L , Wang Y , Yang D , *et al* *Platycodon grandiflorus* ‐ an ethnopharmacolo‐gical, phytochemical and pharmacological review. J Ethnopharmacol. 2015; 164: 147–61. 25666431 10.1016/j.jep.2015.01.052

[55] Ha IJ , Ha YW , Kang M , *et al* Enzymatic transformation of platycosides and one‐step separation of platycodin D by high‐speed countercurrent chromatography. J Sep Sci. 2010; 33: 1916–22. 20533341 10.1002/jssc.200900842

[56] Li T , Xu WS , Wu GS , *et al* Platycodin D induces apoptosis, and inhibits adhesion, migration and invasion in HepG2 hepatocellular carcinoma cells. Asian Pac J Cancer Prev. 2014; 15: 1745–9. 24641402 10.7314/apjcp.2014.15.4.1745

[57] Ahn KS , Hahn BS , Kwack K , *et al* Platycodin D‐induced apoptosis through nuclear factor‐kappaB activation in immortalized keratinocytes. Eur J Pharmacol. 2006; 537: 1–11. 16631160 10.1016/j.ejphar.2006.03.012

[58] Kim TW , Song IB , Lee HK , *et al* Platycodin D, a triterpenoid sapoinin from *Platycodon grandiflorum*, ameliorates cisplatin‐induced nephrotoxicity in mice. Food Chem Toxicol. 2012; 50: 4254–9. 22617354 10.1016/j.fct.2012.05.022

[59] Wu H , Che X , Zheng Q , *et al* Caspases: a molecular switch node in the crosstalk between autophagy and apoptosis. Int J Biol Sci. 2014; 10: 1072–83. 25285039 10.7150/ijbs.9719 PMC4183927

[60] Elmore S . Apoptosis: a review of programmed cell death. Toxicol Pathol. 2007; 35: 495–516. 17562483 10.1080/01926230701320337 PMC2117903

[61] Ferreira CG , Epping M , Kruyt FA , *et al* Apoptosis: target of cancer therapy. Clin Cancer Res. 2002; 8: 2024–34. 12114400

[62] Ouyang L , Shi Z , Zhao S , *et al* Programmed cell death pathways in cancer: a review of apoptosis, autophagy and programmed necrosis. Cell Prolif. 2012; 45: 487–98. 23030059 10.1111/j.1365-2184.2012.00845.x PMC6496669

[63] Kim R , Emi M , Tanabe K . The role of apoptosis in cancer cell survival and therapeutic outcome. Cancer Biol Ther. 2006; 5: 1429–42. 17102590 10.4161/cbt.5.11.3456

[64] Patergnani S , Missiroli S , Marchi S , *et al* Mitochondria‐associated endoplasmic reticulum membranes microenvironment: targeting autophagic and apoptotic pathways in cancer therapy. Front Oncol. 2015; 5: 173. 26284195 10.3389/fonc.2015.00173 PMC4515599

[65] Fuchs Y , Steller H . Programmed cell death in animal development and disease. Cell. 2011; 147: 742–58. 22078876 10.1016/j.cell.2011.10.033 PMC4511103

[66] Volkmann N , Marassi FM , Newmeyer DD , *et al* The rheostat in the membrane: BCL‐2 family proteins and apoptosis. Cell Death Differ. 2014; 21: 206–15. 24162659 10.1038/cdd.2013.153 PMC3890954

[67] Fulda S . Targeting apoptosis for anticancer therapy. Semin Cancer Biol. 2015; 31: 84–8. 24859747 10.1016/j.semcancer.2014.05.002

[68] Hassan M , Watari H , AbuAlmaaty A , *et al* Apoptosis and molecular targeting therapy in cancer. Biomed Res Int. 2014; 2014: 150845. 25013758 10.1155/2014/150845 PMC4075070

[69] Khan M , Bi Y , Qazi JI , *et al* Evodiamine sensitizes U87 glioblastoma cells to TRAIL *via* the death receptor pathway. Mol Med Rep. 2015; 11: 257–62. 25333675 10.3892/mmr.2014.2705

[70] Bake V , Roesler S , Eckhardt I , *et al* Synergistic interaction of Smac mimetic and IFNalpha to trigger apoptosis in acute myeloid leukemia cells. Cancer Lett. 2014; 355: 224–31. 25179908 10.1016/j.canlet.2014.08.040

[71] Mahmood Z , Shukla Y . Death receptors: targets for cancer therapy. Exp Cell Res. 2010; 316: 887–99. 20026107 10.1016/j.yexcr.2009.12.011

[72] Yu JS , Kim AK . Platycodin D induces apoptosis in MCF‐7 human breast cancer cells. J Med Food. 2010; 13: 298–305. 20412017 10.1089/jmf.2009.1226

[73] Chun J , Ha IJ , Kim YS . Antiproliferative and apoptotic activities of triterpenoid saponins from the roots of *Platycodon grandiflorum* and their structure‐activity relationships. Planta Med. 2013; 79: 639–45. 23576176 10.1055/s-0032-1328401

[74] Chun J , Joo EJ , Kang M , *et al* Platycodin D induces anoikis and caspase‐mediated apoptosis *via* p38 MAPK in AGS human gastric cancer cells. J Cell Biochem. 2013; 114: 456–70. 22961809 10.1002/jcb.24386

[75] Chun J , Kim YS . Platycodin D inhibits migration, invasion, and growth of MDA‐MB‐231 human breast cancer cells *via* suppression of EGFR‐mediated Akt and MAPK pathways. Chem Biol Interact. 2013; 205: 212–21. 23867902 10.1016/j.cbi.2013.07.002

[76] Vela L , Marzo I . Bcl‐2 family of proteins as drug targets for cancer chemotherapy: the long way of BH3 mimetics from bench to bedside. Curr Opin Pharmacol. 2015; 23: 74–81. 26079328 10.1016/j.coph.2015.05.014

[77] Fesik SW . Promoting apoptosis as a strategy for cancer drug discovery. Nat Rev Cancer. 2005; 5: 876–85. 16239906 10.1038/nrc1736

[78] Jang YJ , Won JH , Back MJ , *et al* Paraquat induces apoptosis through a mitochondria‐dependent pathway in RAW264.7 cells. Biomol Ther. 2015; 23: 407–13. 10.4062/biomolther.2015.075 PMC4556199 26336579

[79] Gorka M , Godlewski MM , Gajkowska B , *et al* Kinetics of Smac/DIABLO release from mitochondria during apoptosis of MCF‐7 breast cancer cells. Cell Biol Int. 2004; 28: 741–54. 15563396 10.1016/j.cellbi.2004.07.003

[80] Kalimuthu S , Se‐Kwon K . Cell survival and apoptosis signaling as therapeutic target for cancer: marine bioactive compounds. Int J Mol Sci. 2013; 14: 2334–54. 23348928 10.3390/ijms14022334 PMC3587990

[81] Khan M , Yi F , Rasul A , *et al* Alantolactone induces apoptosis in glioblastoma cells *via* GSH depletion, ROS generation, and mitochondrial dysfunction. IUBMB Life. 2012; 64: 783–94. 22837216 10.1002/iub.1068

[82] Khan M , Ding C , Rasul A , *et al* Isoalantolactone induces reactive oxygen species mediated apoptosis in pancreatic carcinoma PANC‐1 cells. Int J Biol Sci. 2012; 8: 533–47. 22532787 10.7150/ijbs.3753 PMC3334669

[83] Srinivasula SM , Datta P , Fan XJ , *et al* Molecular determinants of the caspase‐promoting activity of Smac/DIABLO and its role in the death receptor pathway. J Biol Chem. 2000; 275: 36152–7. 10950947 10.1074/jbc.C000533200

[84] Xu C , Sun G , Yuan G , *et al* Effects of platycodin D on proliferation, apoptosis and PI3K/Akt signal pathway of human glioma U251 cells. Molecules. 2014; 19: 21411–23. 25532840 10.3390/molecules191221411 PMC6270900

[85] Gorlach A , Dimova EY , Petry A , *et al* Reactive oxygen species, nutrition, hypoxia and diseases: problems solved? Redox Biol. 2015; 6: 372–85. 26339717 10.1016/j.redox.2015.08.016 PMC4565025

[86] Gorlach A , Bertram K , Hudecova S , *et al* Calcium and ROS: a mutual interplay. Redox Biol. 2015; 6: REDOXD1500102. 10.1016/j.redox.2015.08.010 PMC4556774 26296072

[87] Trachootham D , Alexandre J , Huang P . Targeting cancer cells by ROS‐mediated mechanisms: a radical therapeutic approach? Nat Rev Drug Discov. 2009; 8: 579–91. 19478820 10.1038/nrd2803

[88] Hong YH , Uddin MH , Jo U , *et al* ROS accumulation by PEITC selectively kills ovarian cancer cells *via* UPR‐mediated apoptosis. Front Oncol. 2015; 5: 167. 26284193 10.3389/fonc.2015.00167 PMC4517521

[89] Zhu L , Ren L , Chen Y , *et al* Redox status of high‐mobility group box 1 performs a dual role in angiogenesis of colorectal carcinoma. J Cell Mol Med. 2015; 19: 2128–35. 26099505 10.1111/jcmm.12577 PMC4568917

[90] Wei C , Xiao Q , Kuang X , *et al* Fucoidan inhibits proliferation of the SKM‐1 acute myeloid leukaemia cell line *via* the activation of apoptotic pathways and production of reactive oxygen species. Mol Med Rep. 2015; 12: 6649–55. 26324225 10.3892/mmr.2015.4252 PMC4626197

[91] Seo KH , Ryu HW , Park MJ , *et al* Mangosenone F, a furanoxanthone from *Garciana mangostana*, induces reactive oxygen species‐mediated apoptosis in lung cancer cells and decreases xenograft tumor growth. Phytother Res. 2015; 29: 1753–60. 26310849 10.1002/ptr.5428

[92] Shin DY , Kim GY , Li W , *et al* Implication of intracellular ROS formation, caspase‐3 activation and Egr‐1 induction in platycodon D‐induced apoptosis of U937 human leukemia cells. Biomed Pharmacother. 2009; 63: 86–94. 18804340 10.1016/j.biopha.2008.08.001

[93] Yu JS , Kim AK . Platycodin D induces reactive oxygen species‐mediated apoptosis signal‐regulating kinase 1 activation and endoplasmic reticulum stress response in human breast cancer cells. J Med Food. 2012; 15: 691–9. 22784044 10.1089/jmf.2011.2024

[94] Benada J , Macurek L . Targeting the checkpoint to kill cancer cells. Biomolecules. 2015; 5: 1912–37. 26295265 10.3390/biom5031912 PMC4598780

[95] Diaz‐Moralli S , Tarrado‐Castellarnau M , Miranda A , *et al* Targeting cell cycle regulation in cancer therapy. Pharmacol Ther. 2013; 138: 255–71. 23356980 10.1016/j.pharmthera.2013.01.011

[96] Lee CT , Huang YW , Yang CH , *et al* Drug delivery systems and combination therapy by using vinca alkaloids. Curr Top Med Chem. 2015; 15: 1491–500. 25877096 10.2174/1568026615666150414120547 PMC4997956

[97] Khanna C , Rosenberg M , Vail DM . A review of paclitaxel and novel formulations including those suitable for use in dogs. J Vet Intern Med. 2015; 29: 1006–12. 26179168 10.1111/jvim.12596 PMC4895360

[98] Mukhtar E , Adhami VM , Mukhtar H . Targeting microtubules by natural agents for cancer therapy. Mol Cancer Ther. 2014; 13: 275–84. 24435445 10.1158/1535-7163.MCT-13-0791 PMC3946048

[99] Klute K , Nackos E , Tasaki S , *et al* Microtubule inhibitor‐based antibody‐drug conjugates for cancer therapy. Onco Targets Ther. 2014; 7: 2227–36. 25506226 10.2147/OTT.S46887 PMC4259504

[100] Qin H , Du X , Zhang Y , *et al* Platycodin D, a triterpenoid saponin from *Platycodon grandiflorum*, induces G2/M arrest and apoptosis in human hepatoma HepG2 cells by modulating the PI3K/Akt pathway. Tumour Biol. 2014; 35: 1267–74. 24048756 10.1007/s13277-013-1169-1

[101] Kim MO , Moon DO , Choi YH , *et al* Platycodin D induces mitotic arrest *in vitro*, leading to endoreduplication, inhibition of proliferation and apoptosis in leukemia cells. Int J Cancer. 2008; 122: 2674–81. 18351645 10.1002/ijc.23442

[102] Kim MO , Moon DO , Choi YH , *et al* Platycodin D induces apoptosis and decreases telomerase activity in human leukemia cells. Cancer Lett. 2008; 261: 98–107. 18093727 10.1016/j.canlet.2007.11.010

[103] Lee SK , Park KK , Kim HJ , *et al* Platycodin D blocks breast cancer‐induced bone destruction by inhibiting osteoclastogenesis and the growth of breast cancer cells. Cell Physiol Biochem. 2015; 36: 1809–20. 26184636 10.1159/000430152

[104] Li Z , Wang J , Yang X . Functions of autophagy in pathological cardiac hypertrophy. Int J Biol Sci. 2015; 11: 672–8. 25999790 10.7150/ijbs.11883 PMC4440257

[105] Sui X , Kong N , Ye L , *et al* p38 and JNK MAPK pathways control the balance of apoptosis and autophagy in response to chemotherapeutic agents. Cancer Lett. 2014; 344: 174–9. 24333738 10.1016/j.canlet.2013.11.019

[106] Panda PK , Mukhopadhyay S , Das DN , *et al* Mechanism of autophagic regulation in carcinogenesis and cancer therapeutics. Semin Cell Dev Biol. 2015; 39: 43–55. 25724561 10.1016/j.semcdb.2015.02.013

[107] Hasima N , Ozpolat B . Regulation of autophagy by polyphenolic compounds as a potential therapeutic strategy for cancer. Cell Death Dis. 2014; 5: e1509. 25375374 10.1038/cddis.2014.467 PMC4260725

[108] Law BY , Chan WK , Xu SW , *et al* Natural small‐molecule enhancers of autophagy induce autophagic cell death in apoptosis‐defective cells. Sci Rep. 2014; 4: 5510. 24981420 10.1038/srep05510 PMC4076737

[109] Lee YJ , Hah YJ , Kang YN , *et al* The autophagy‐related marker LC3 can predict prognosis in human hepatocellular carcinoma. PLoS ONE. 2013; 8: e81540. 24282606 10.1371/journal.pone.0081540 PMC3839913

[110] Wu SY , Sung PJ , Chang YL , *et al* Heteronemin, a spongean sesterterpene, induces cell apoptosis and autophagy in human renal carcinoma cells. Biomed Res Int. 2015; 2015: 738241. 26090440 10.1155/2015/738241 PMC4450260

[111] Li T , Tang ZH , Xu WS , *et al* Platycodin D triggers autophagy through activation of extracellular signal‐regulated kinase in hepatocellular carcinoma HepG2 cells. Eur J Pharmacol. 2015; 749: 81–8. 25592318 10.1016/j.ejphar.2015.01.003

[112] Zhao R , Chen M , Jiang Z , *et al* Platycodin‐D induced autophagy in non‐small cell lung cancer cells *via* PI3K/Akt/mTOR and MAPK signaling pathways. J Cancer. 2015; 6: 623–31. 26078792 10.7150/jca.11291 PMC4466411

[113] Siveen KS , Sikka S , Surana R , *et al* Targeting the STAT3 signaling pathway in cancer: role of synthetic and natural inhibitors. Biochim Biophys Acta. 2014; 1845: 136–54. 24388873 10.1016/j.bbcan.2013.12.005

[114] Jayapal SR , Lee KL , Ji P , *et al* Down‐regulation of Myc is essential for terminal erythroid maturation. J Biol Chem. 2010; 285: 40252–65. 20940306 10.1074/jbc.M110.181073 PMC3001006

[115] Bretones G , Delgado MD , Leon J . Myc and cell cycle control. Biochim Biophys Acta. 2015; 1849: 506–16. 24704206 10.1016/j.bbagrm.2014.03.013

[116] Mukhopadhyay A , Banerjee S , Stafford LJ , *et al* Curcumin‐induced suppression of cell proliferation correlates with down‐regulation of cyclin D1 expression and CDK4‐mediated retinoblastoma protein phosphorylation. Oncogene. 2002; 21: 8852–61. 12483537 10.1038/sj.onc.1206048

[117] Lattanzio R , Marchisio M , La Sorda R , *et al* Overexpression of activated phospholipase C gamma1 is a risk factor for distant metastases in T1‐T2, N0 breast cancer patients undergoing adjuvant chemotherapy. Int J Cancer. 2013; 132: 1022–31. 22847294 10.1002/ijc.27751

[118] Luan X , Gao YG , Guan YY , *et al* Platycodin D inhibits tumor growth by antiangiogenic activity *via* blocking VEGFR2‐mediated signaling pathway. Toxicol Appl Pharmacol. 2014; 281: 118–24. 25250884 10.1016/j.taap.2014.09.009

[119] Prabhu L , Mundade R , Korc M , *et al* Critical role of NF‐kappaB in pancreatic cancer. Oncotarget. 2014; 5: 10969–75. 25473891 10.18632/oncotarget.2624 PMC4294354

[120] Verstrepen L , Beyaert R . Receptor proximal kinases in NF‐kappaB signaling as potential therapeutic targets in cancer and inflammation. Biochem Pharmacol. 2014; 92: 519–29. 25449604 10.1016/j.bcp.2014.10.017

[121] Kim NH , Pham NB , Quinn RJ , *et al* The small molecule R‐(‐)‐beta‐O‐methylsynephrine binds to nucleoporin 153 kDa and inhibits angiogenesis. Int J Biol Sci. 2015; 11: 1088–99. 26221075 10.7150/ijbs.10603 PMC4515819

[122] Zhao Y , Adjei AA . Targeting angiogenesis in cancer therapy: moving beyond vascular endothelial growth factor. Oncologist. 2015; 20: 660–73. 26001391 10.1634/theoncologist.2014-0465 PMC4571783

[123] Kang FB , Wang L , Jia HC , *et al* B7‐H3 promotes aggression and invasion of hepatocellular carcinoma by targeting epithelial‐to‐mesenchymal transition *via* JAK2/STAT3/Slug signaling pathway. Cancer Cell Int. 2015; 15: 45. 25908926 10.1186/s12935-015-0195-z PMC4407415

[124] Alias C , Rocchi L , Ribatti D , *et al* MMPs and angiogenesis affect the metastatic potential of a human vulvar leiomyosarcoma cell line. J Cell Mol Med. 2015; 19: 2098–107. 26010680 10.1111/jcmm.12565 PMC4568914

[125] Guan X . Cancer metastases: challenges and opportunities. Acta Pharmaceutica Sinica B. 2015; 5: 402–418. 26579471 10.1016/j.apsb.2015.07.005 PMC4629446

[126] Tang ZH , Li T , Gao HW , *et al* Platycodin D from Platycodonis Radix enhances the anti‐proliferative effects of doxorubicin on breast cancer MCF‐7 and MDA‐MB‐231 cells. Chin Med. 2014; 9: 16. 24982689 10.1186/1749-8546-9-16 PMC4075934

